# A Comparison of Surgical and Functional Outcomes in Prostate Cancer Patients with Overweight and Obesity Participating in a Presurgical Weight Loss Trial

**DOI:** 10.3390/cancers17091496

**Published:** 2025-04-29

**Authors:** Madeline F. Morgan, Andrew D. Frugé, Wendy Demark-Wahnefried, Jeffrey W. Nix, Soroush Rais-Bahrami

**Affiliations:** 1 Department of Medicine, University of Alabama at Birmingham (UAB) Heersink School of Medicine, Birmingham, AL 35294, USA; 2 College of Nursing, Auburn University, Auburn, AL 36849, USA; 3 O’Neal Comprehensive Cancer Center at UAB, Birmingham, AL 35294, USA; 4 Department of Nutrition Sciences, UAB, Birmingham, AL 35233, USA; 5 Department of Radiology, UAB, Birmingham, AL 35233, USA; 6 Department of Urology, UAB, Birmingham, AL 35233, USA

**Keywords:** abdominal adiposity, functional outcomes, obesity, prostate cancer, surgical complications, surgical outcomes, total body fat mass

## Abstract

Background/Objectives: Obesity and abdominal adiposity are associated with worse surgical and functional outcomes in prostate cancer (PCa) patients. This exploratory study assessed whether reductions in total body fat mass (TFM) among overweight and obese PCa patients enrolled in a diet and exercise weight loss intervention prior to robotic-assisted radical prostatectomy (RARP) improved outcomes. Methods: In this secondary analysis of an NIH-funded randomized controlled trial (NCT01886677) conducted 2012–2015, twenty-nine patients with newly diagnosed, pathology-confirmed PCa who participated and underwent RARP were evaluated for percent change in TFM and divided into High Fat Losers who lost ≥1% TFM per week and Low Fat Losers who lost <1% TFM per week. High versus Low Fat Losers were compared on operative time (OT), estimated blood loss (EBL), length of hospital stay (LOS), incidence of surgical or postoperative complications, and incontinence and impotence scores at first postoperative follow-up. Results: There was a statistically significant difference between High versus Low Fat Losers with respect to overall complications (*p* = 0.027); 28.6% of High Fat Losers experienced one or more complications by first postoperative follow-up, compared to 73.3% of Low Fat Losers. However, no differences were observed for each individual complication analyzed, or with respect to OT, EBL, LOS, or incontinence or impotence scores. Conclusions and Relevance: Findings implicate the potential benefit of healthy weight loss as an adjunct to surgery, and support the need for larger trials to elucidate a clearer relationship between improvements in body composition and effects on specific surgical complications and functional outcomes.

## Simple Summary

Question: Do presurgical decreases in total body fat mass (TFM) among prostate cancer patients with elevated body mass index affect surgical and functional outcomes of robotic-assisted radical prostatectomy (RARP)? Findings: Participants with greater losses of TFM prior to RARP experienced fewer surgical complications overall (*p* = 0.027); 28.6% of High Fat Losers experienced one or more complications by first postoperative follow-up, compared to 73.3% of Low Fat Losers. Meaning: Healthy weight loss and improvement in body composition may be helpful adjuncts to surgery in obese and overweight prostate cancer patients, but further research is needed to elucidate effects on specific complications and functional outcomes in larger samples of patients.

## 1. Introduction

Numerous studies support the association between obesity (body mass index (BMI) ≥30 kg/m^2^) and more aggressive prostate cancer (PCa) [1], with higher disease-specific and overall mortality reported among patients with obesity [2]. Some studies implicate the additional role of abdominal adiposity, linking increased disease aggressiveness to higher waist circumference (WC) [3] and abdominal visceral fat [4]—a phenotype associated with higher levels of growth factors and hormones implicated in PCa biology [4]. Additionally, obesity and abdominal adiposity have demonstrated detrimental effects on various surgical parameters in PCa patients. Obesity is linked to increased operative times (OT) and estimated blood loss (EBL) in both open and robotic-assisted radical prostatectomy (RARP) [1,5,6]. Similarly, high periprostatic fat area is associated with longer OT [7], and high WC is associated with increased intraoperative complications [8]. Postoperatively, obesity is linked to greater length of hospital stays (LOS) [5] and increased incidence of vesicourethral stricture and wound infection [9]. Worse functional outcomes, including slower recovery of continence and erectile function, are reported in patients with obesity [1,6,9].

While many studies quantify the adverse effects of obesity and abdominal adiposity on surgical and functional outcomes in PCa patients, none have explored whether reduced adiposity among overweight and obese patients correlates with improved outcomes. Additionally, no studies have assessed outcomes relative to precise measures of body composition, such as total body fat mass (TFM), as measured via dual energy X-ray absorptiometry (DXA). More research is needed to validate the utility of weight loss and improved body composition as adjuncts to surgical management of PCa patients. This exploratory study assessed the impact of presurgical decreases in TFM on surgical and functional outcomes of RARP, specifically evaluating whether greater TFM loss correlated with reduced OT, EBL, LOS, and intra- or postoperative complications, or with improved continence and erectile function at first postoperative follow-up.

## 2. Materials and Methods

### 2.1. Design and Participants

This study is a secondary analysis of NCT01886677 [10], an NIH-funded randomized controlled trial evaluating the effects of a presurgical calorie-restricted diet and exercise intervention (aiming for ~1 kg of weight loss/week between diagnosis and surgery) on body composition, tumor characteristics, and serum biomarkers among overweight or obese PCa patients. Participants included 40 men with overweight (BMI 25–29.9) or obesity (BMI ≥ 30) and newly-diagnosed, pathology-confirmed PCa who elected initial treatment with RARP at the University of Alabama at Birmingham or Urology Centers of Alabama. The present study analyzed additional surgical and postoperative data to determine whether improvements in body composition affected surgical and functional outcomes. Thirty-two patients underwent surgery and provided verbal consent for chart review of operative/hospital notes, pathology reports, and first postoperative visit notes.

### 2.2. Measures

Anthropometric measures obtained at intervention baseline and one day prior to surgery included TFM measured by DXA, weight, height, and BMI. Patient demographics, diabetic and smoking status, and baseline PSA were recorded. Operative and hospital notes were reviewed for OT, EBL, and LOS by a physician (MFM) who was blinded with regard to fat mass loss. Intraoperative or postoperative complications also were recorded and tallied; these included the following: rectal, bladder neck, nerve compression, or acute kidney injury; anastomosis leak; vesicourethral stricture; wound or urinary tract infection; postoperative bleed; scrotal wall edema/hydrocele; psoas abscess, small bowel obstruction, pulmonary complications (pneumonia, pulmonary edema), and deep vein thrombosis. Each patient received a net complication score (0: none; 1: any complications). Patients received an incontinence score (0: none; 1: mild; 2: moderate; 3: severe) and impotence score (0: none; 1: partial; 2: full impotence) based on review of first postoperative visit notes. Gleason score and pathologic T-stage and N-stage were obtained from pathology reports. Times between intervention baseline, surgery, and first postoperative visit were recorded.

### 2.3. Statistics

Statistical analyses were conducted in SPSS v29 (IBM Corp, Armonk, NY, USA). Continuous variables were assessed for normality using the Shapiro–Wilk Test. Given the non-normal distribution of several variables and small sample size, nonparametric tests were employed. Rates of percent change in TFM for each patient were calculated by dividing the percent change from intervention baseline to surgery by the number of weeks on study. Participants were divided into two groups at the median: High Fat Losers with ≥1% TFM loss per week and Low Fat Losers with <1% TFM loss per week, resulting in the allocation of three men from the control arm to High Fat Losers group and three men from the intervention arm to the High Fat Losers group. High versus Low Fat Losers were compared on demographics, baseline characteristics, and the intraoperative, postoperative, and functional outcome measures above, using Fisher’s exact test and chi-square analysis for categorical variables and Mann–Whitney U tests for continuous variables (*p* < 0.05 was considered significant).

## 3. Results

Of the thirty-two total participants, one was excluded due to unavailability of DXA data and two were excluded for receiving open radical prostatectomy, for a total of twenty-nine subjects.

There were no significant differences between High versus Low Fat Losers in terms of age, race, diabetic or smoking status, PSA, or baseline weight, height, or BMI (Table 1), or in terms of Gleason score (*p* = 0. 210), T-stage (*p* = 0.500), or N-stage (*p* = 0.185) on radical prostatectomy pathology.

Participants who lost more versus less body fat were significantly less likely to have any complications, *p* = 0.027 (Fisher’s exact test). Among High Fat Losers, four (28.6%) had one or more complications and ten (71.4%) had none; among Low Fat Losers, eleven (73.3%) had one or more complications and four (26.7%) had none. Results of analyses of other surgical and functional outcomes are shown in Table 2. Although there was a significant difference in the incidence of overall complications, there were no significant differences with respect to each individual complication analyzed, with anastomosis leak being the most common (n = 5), or with respect to OT, EBL, LOS, or incontinence or impotence scores.

## 4. Discussion

While many studies describe the detrimental effects of obesity and abdominal adiposity on the surgical and functional outcomes of prostatectomy, this exploratory study is the first to evaluate the relationship between reductions in adiposity measures among overweight and obese PCa patients and possible improvements in outcomes. Our finding that patients with greater TFM loss were less likely to experience complications supports the potential benefit of intentional weight loss via diet and exercise as an adjunct to surgery, and suggests that improvement in body composition via a reduction in total body fat may be instrumental. However, the low incidence of each individual complication prevented the discovery of complications most affected by TFM loss, while also reducing potential clinical significance. The limited sample size of this exploratory study also cautions the generalizability of findings, and a need to view results strictly as hypothesis-generating.

## 5. Conclusions

Larger prospective studies of longer-duration weight loss interventions among surgical PCa patients are needed in order to elucidate a clearer relationship between changes in anthropometric measures and clinical benefit with respect to surgical outcomes.

## Figures and Tables

**Table 1:** Baseline characteristics and demographics of prostate cancer patients participating in the weight loss trial.

Characteristic	High-Fat Losers * (n = 14)	Low-Fat Losers † (n = 15)	*p*-Value
Age (years), Mean (s.d.)	59.4 (6.8)	60.4 (6.8)	0.621
Race African American, n (%) Non-Hispanic White, n (%)	2 (14.3) 12 (85.7)	7 (46.7) 8 (53.3)	0.109
Diabetes, n (%)	3 (21.4)	4 (26.7)	1.000
Current smoker, n (%)	1 (7.1)	2 (13.3)	1.000
Weight (kg), Mean (s.d.)	97.0 (12.8)	95.7 (13.5)	0.949
Height (cm), Mean (s.d.)	176.2 (4.8)	177.6 (7.4)	0.451
BMI (kg/m^2^), Mean (s.d.)	31.3 (4.5)	30.2 (2.6)	0.621
PSA, Mean (s.d.)	7.2 (3.6)	7.6 (3.7)	0.683

* Participants with ≥1% loss of total fat mass/week. † Participants with <1% loss of total fat mass/week.

**Table 2:** Surgical parameters, complications, and functional outcomes among High versus Low Fat Losers.

Outcomes	High Fat Losers (n = 14)	Low Fat Losers (n = 15)	*p*-Value
Percent Δ in TFM/week (%), Mean (s.d.)	−1.55 (0.68)	−0.24 (0.58)	<0.001
Operative time (minutes), Mean (s.d.)	201.7 (68.5)	206.4 (78.2)	0.505
Estimated blood loss (mL), Mean (s.d.)	156.7 (156.8)	171.4 (123.6)	0.477
Length of hosp. stay (days), Mean (s.d.)	1.9 (2.1)	1.7 (1.6)	0.429
Anastomosis leak, yes-n (%)	2 (14.3)	3 (20.0)	1.000
One or more complication(s), * yes-n (%)	4 (28.6)	11 (73.3)	0.027
Incontinence score, † Mean (s.d.)	1.3 (1.1)	1.6 (0.7)	0.315
Impotence score, † Mean (s.d.)	0.9 (0.7)	1.3 (0.6)	0.284

* Complications included rectal injury, bladder neck injury, anastomosis leak, vesicourethral stricture, wound infection, postoperative bleed, scrotal wall edema/hydrocele, psoas abscess, nerve compression injury, small bowel obstruction, acute kidney injury, pulmonary complications (pneumonia, pulmonary edema), deep vein thrombosis, and urinary tract infection. † Chi-square tests conducted on scores ranging from 0–3 (incontinence) and 0–2 (impotence).

## Data Availability

Data are available upon reasonable request from the corresponding author.

## Abbreviations

The following abbreviations are used in this manuscript:

BMI	Body Mass Index
DXA	Dual Energy X-ray Absorptiometry
EBL	Estimated Blood Loss
LOS	Length of Hospital Stay
NIH	National Institutes of Health
OT	Operative Time
PCa	Prostate Cancer
PSA	Prostate-Specific Antigen
RARP	Robotic-Assisted Radical Prostatectomy
TFM	Total Body Fat Mass
WC	Waist Circumference
